# Glutathione peroxidase 3 localizes to the epithelial lining fluid and the extracellular matrix in interstitial lung disease

**DOI:** 10.1038/srep29952

**Published:** 2016-07-20

**Authors:** Andrea C. Schamberger, Herbert B. Schiller, Isis E. Fernandez, Martina Sterclova, Katharina Heinzelmann, Elisabeth Hennen, Rudolf Hatz, Jürgen Behr, Martina Vašáková, Matthias Mann, Oliver Eickelberg, Claudia A. Staab-Weijnitz

**Affiliations:** 1 Comprehensive Pneumology Center, Helmholtz Zentrum München, Member of the German Center of Lung Research (DZL), Munich, Germany; 2 Department of Proteomics and Signal Transduction, Max-Planck Institute of Biochemistry, Martinsried, Germany; 3 Department of Respiratory Medicine of the 1st Medical School, Charles University and Thomayer Hospital, Prague, Czech republic; 4 Thoraxchirurgisches Zentrum, Klinik für Allgemeine-, Viszeral-, Transplantations-, Gefäß- und Thoraxchirurgie, Klinikum Großhadern, Ludwig-Maximilians-Universität, Munich, Germany; 5 Asklepios Fachkliniken München-Gauting, Munich, Germany; 6 Medizinische Klinik und Poliklinik V, Klinikum der Ludwig-Maximilians-Universität, Member of the German Center for Lung Research (DZL), Munich, Germany

## Abstract

Aberrant antioxidant activity and excessive deposition of extracellular matrix (ECM) are hallmarks of interstitial lung diseases (ILD). It is known that oxidative stress alters the ECM, but extracellular antioxidant defence mechanisms in ILD are incompletely understood. Here, we extracted abundance and detergent solubility of extracellular antioxidant enzymes from a proteomic dataset of bleomycin-induced lung fibrosis in mice and assessed regulation and distribution of glutathione peroxidase 3 (GPX3) in murine and human lung fibrosis. Superoxide dismutase 3 (Sod3), Gpx3, and Gpx activity were increased in mouse BALF during bleomycin-induced lung fibrosis. In lung tissue homogenates, Gpx3, but not Sod3, was upregulated and detergent solubility profiling indicated that Gpx3 associated with ECM proteins. Immunofluorescence analysis showed that Gpx3 was expressed by bronchial epithelial cells and interstitial fibroblasts and localized to the basement membrane and interstitial ECM in lung tissue. As to human ILD samples, BALF of some patients contained high levels of GPX3, and GPX3 was upregulated in lung homogenates from IPF patients. GPX3 expression in primary human bronchial epithelial cells and lung fibroblasts was downregulated by TNF-α, but more variably regulated by TGF-β1 and menadione. In conclusion, the antioxidant enzyme GPX3 localizes to lung ECM and is variably upregulated in ILD.

The collective term interstitial lung disease (ILD) comprises various lung conditions which are characterized by thickening of the alveolar walls by inflammation or fibrosis. ILD can be divided into three subcategories: Exposure-related ILD, systemic disease-related ILD, and ILD of unknown cause. For example, hypersensitivity pneumonitis (HP), also referred to as extrinsic allergic alveolitis (EAA), is caused by inhalation of various organic antigens and can, if exposure is chronic, lead to severe impairment of lung function and distortion of lung structure reminiscent of idiopathic pulmonary fibrosis (IPF), the most aggressive ILD^1^,^2^. Smoking-related interstitial fibrosis (SRIF) is an ILD common in cigarette smokers which is characterized by alveolar septa fibrosis and minimal inflammation^3^. In contrast, sarcoidosis is a systemic primarily inflammatory disease which can involve multiple organs, but mostly affects the lung. Typically, nodules of inflamed tissue (so-called granulomas) form in the affected organs which may resolve without irreversible damage^4^. In 20–25% of the patients, however, pulmonary fibrosis occurs leading to permanent lung dysfunction^5^. Finally, idiopathic pulmonary fibrosis (IPF) is a particularly aggressive and progressive ILD pathogenically based on an aberrant fibroproliferative wound healing response following multiple alveolar lesions, with a 5-yr survival of 30%^6^,^7^.

Several lines of evidence support the involvement of oxidative stress in fibrotic lung disease^8^,^9^. For instance, markers of increased oxidative stress have been detected in exhaled air and bronchoalveolar lavage fluid (BALF) of patients suffering from IPF, sarcoidosis and HP^10^,^11^,^12^,^13^,^14^. Furthermore, depletion of glutathione, the most abundant low-molecular-weight antioxidant, has been reported in the epithelial lining fluid (ELF) of IPF, sarcoidosis, and HP patients^14^,^15^,^16^,^17^,^18^. Finally, numerous endogenous and exogenous agents implicated in the aetiology of pulmonary fibrosis cause levels of reactive oxygen species (ROS) to increase. Extrinsic sources include cigarette smoke, asbestos, silica, and bleomycin, all well-known risk factors for pulmonary fibrosis. Endogenous ROS sources include superoxide and hydrogen peroxide-producing phagocytic cells, but also intra- and extracellular enzymatic systems which produce ROS, as *e.g.* members of the NADPH oxidase (NOX) family, the mitochondrial electron transport chain, or extracellular lysyl oxidase activity in collagen crosslinking^19^,^20^,^21^,^22^.

Excessive deposition of extracellular matrix (ECM) and lung tissue remodelling is a central characteristic of fibrotic disorders and the ECM has been attributed a key role in the progressive nature of IPF^23^. Importantly, the composition of the ECM is affected by oxidative stress^24^. Several *in vivo* studies in mouse models of lung fibrosis have shown that ECM components as *e.g.* collagen, heparan sulphate, syndecan, and hyaluronic acid are increasingly fragmented or shed from the cell surface in response to oxidative stress^25^,^26^,^27^,^28^. In line with these observations, increased levels of collagen III, hyaluronic acid, and syndecan-1 have also been reported in BALF from IPF and HP patients^24^,^27^,^29^,^30^.

A number of extracellular antioxidant proteins have been described to localize to the ECM of the lung, suggesting a potential protective role in presence of oxidative stress^31^, in particular, glutathione peroxidase 3 (GPX3) and extracellular superoxide dismutase (EC-SOD or SOD3)^26^,^27^,^28^,^32^,^33^. While the role of EC-SOD in pulmonary fibrosis has received considerable attention^34^, the regulation and distribution of GPX3 in this context has, to our knowledge, not been studied.

In the present study, we sought to assess localization, expression, and regulation of GPX3 expression in normal and fibrotic lung. We analysed Gpx3 levels and Gpx activity in the time course of bleomycin-induced lung fibrosis and studied tissue distribution in normal and fibrotic mouse lungs. Levels of GPX3 were measured in human BALF and total tissue lysates from ILD patients and localization of GPX3 assessed in human lung sections. Finally, regulation of GPX3 by representative proinflammatory and profibrotic mediators as well as menadione as inducer of oxidative stress was assessed in differentiated primary human bronchial epithelial cells (phBEC) as well as primary human lung fibroblasts (phLF).

## Results

### Gpx3 is upregulated in BALF and tissue in the mouse model of bleomycin-induced pulmonary fibrosis

**Figure 1:**
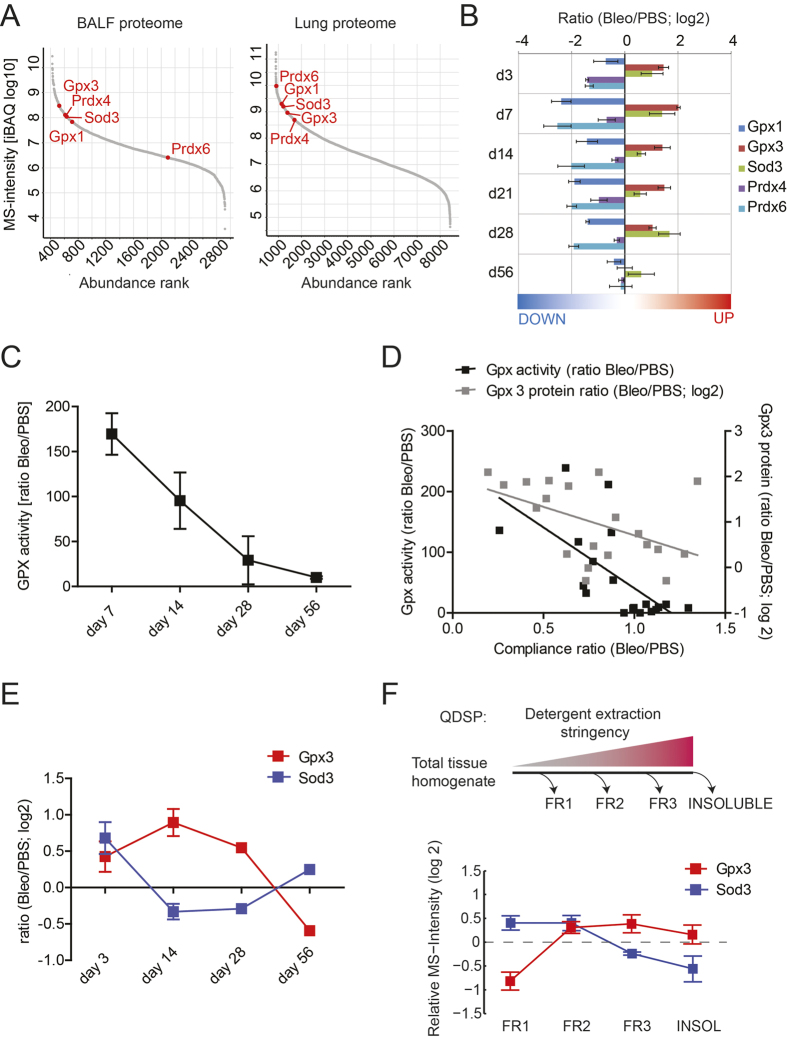
Of all antioxidant proteins known to associate with the extracellular compartment, only Gpx3 is upregulated both in BALF and tissue during bleomycin-induced lung fibrosis and enriched in the matrisome fraction. (**A**) Label-free proteome quantification by iBAQ shows relative abundance of the proteins in BALF (left-hand panel) and the lung proteome (right-hand panel) under basal conditions, *i.e.* in control mice. (**B**) Bar graph showing regulation of major lung antioxidant proteins during bleomycin-induced lung fibrosis. (**C**) Change of selenium-dependent Gpx activity in BALF during bleomycin-induced lung fibrosis. Activity ratios (i.e. Gpx activity in the Bleomycin group relative to PBS control) are shown for each time point. (**D**) Correlation of normalized compliance values with normalized Gpx3 protein levels as extracted from Schiller *et al*.^35^ (grey boxes) and with Gpx activity (black boxes, data from **C**) in the time course of bleomycin-induced lung fibrosis. Here, data from all time points were used and correlated. (**E**) Regulation of Gpx3 and Sod3 in tissue during bleomycin-induced lung fibrosis. (**F**) Quantitative Detergent Solubility Profiling (QDSP) shows enrichment of Gpx3, but not Sod3, in the insoluble fractions. The depicted data is derived from the bleomycin group at day 14^35^. Data in A, B, E and F were extracted from a recently acquired proteomic data set by Schiller *et al*.^35^.

Initially, in order to assess expression and regulation of lung-abundant extracellular antioxidant proteins, we extracted the relevant data from a previously published proteomic study on bleomycin-induced pulmonary fibrosis^35^. Lung-abundant antioxidant proteins with known extracellular location include Gpx1^36^, Gpx3^32^,^33^, Sod3^37^, peroxiredoxin IV (Prdx4)^31^, and peroxiredoxin VI (Prdx6)^38^. All of these, except for Prdx6, were highly abundant in BALF of control mice under basal conditions (Fig. 1A, left-hand panel) and Gpx3 and Sod3 were additionally upregulated in BALF during bleomycin-induced lung fibrosis (Fig. 1B). As protein abundance of Gpx3 in BALF under normal conditions was substantially higher than for Gpx1 (Fig. 1A, left-hand panel, appr. 5 times higher), we reasoned that selenium-dependent Gpx activity in BALF should reflect GPX3 activity. In agreement, selenium-dependent Gpx activity was significantly increased 7 and 14 days after bleomycin instillation and returned to baseline thereafter (Fig. 1C). Finally, both BALF Gpx3 levels as well as Gpx activity in BALF showed anti-correlation with lung function (Spearman r = −0.4135, p = 0.0699 for Gpx3 protein; Spearman r = −0.7152, p = 0.0006 for Gpx activity; Fig. 1D).

In lung tissue, all antioxidant proteins assessed belonged to the quartile of proteins with the highest abundance and, here, contrary to BALF, Prdx6 displayed the highest levels (Fig. 1A, right-hand panel). In response to bleomycin, Gpx3 was upregulated also in tissue, albeit to a lesser extent compared to the increase in BALF, while Sod3 was largely unchanged, except during initial acute lung injury (Fig. 1E). Furthermore, in contrast to Sod3, tissue Gpx3 was predominantly detected in the insoluble protein fractions, as judged from quantitative detergent solubility profiling (QDSP) generated by Schiller *et al*.^35^ (Fig. 1F). Notably, these insoluble fractions are highly enriched for ECM and matrisome-associated proteins^35^,^39^.

### In mouse lung, Gpx3 is expressed by bronchial epithelial cells and lung fibroblasts and localizes to the ECM

**Figure 2:**
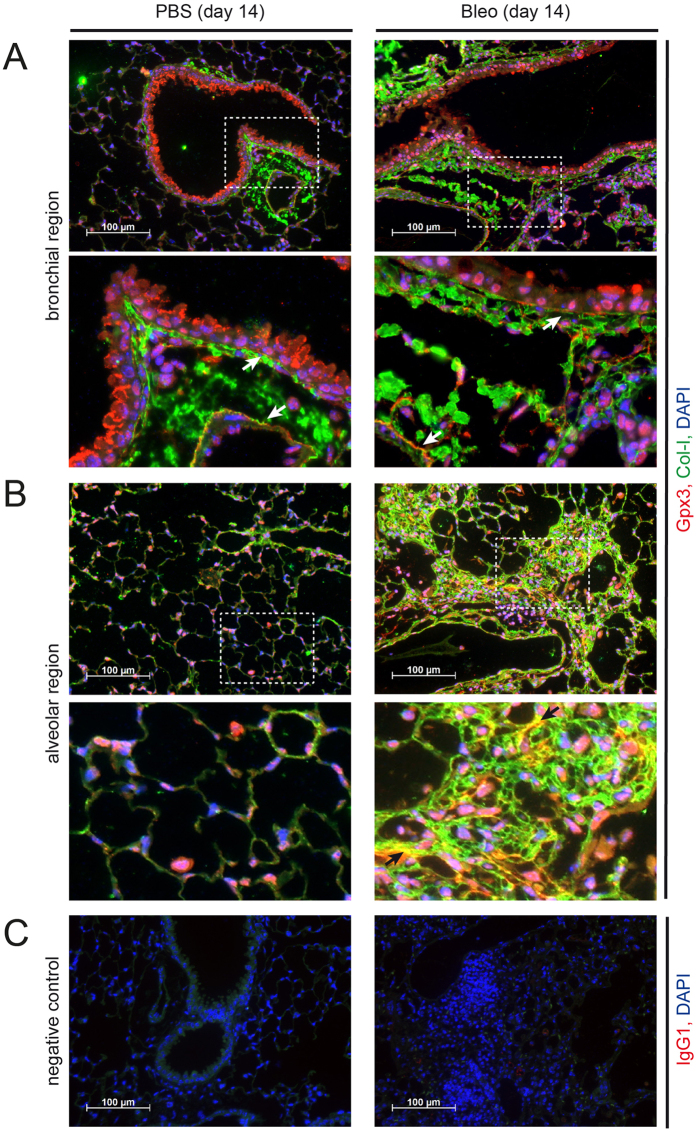
Gpx3 is secreted by bronchial epithelial cells and localizes to ECM structures in control (PBS) and bleomycin (Bleo) instilled mouse lung. Immunofluorescence analysis of PBS (left-hand panels) and Bleo (right-hand panels) mouse lungs at day 14 after instillation. Representative images with Gpx3 (red), Col-I (green), and DAPI (blue) are shown from bronchial (**A**) and alveolar regions (**B**) together with an isotype control for Gpx3 staining (**C**). Scale bar: 100 μm. Arrows in the higher magnification inserts indicate colocalization of Gpx3 with ECM structures: Subendothelial and subepithelial basement membrane (**A**) and interstitial ECM (**B**, only right-hand panel).

Immunofluorescent stainings showed that, in normal mouse lungs, Gpx3 was mostly secreted by bronchial epithelial cells, where clear vesicular staining was observed (Fig. 2A, left-hand panels). In addition, we detected localization to the subendothelial and subepithelial basement membrane (Fig. 2A, left-hand panel, higher magnification insert). Upon development of lung fibrosis following tracheal instillation of bleomycin, Gpx3 expression was observed in many more cells including bronchial epithelial cells and interstitial fibroblasts (Fig. 2A,B, right-hand panels). Localization to the subendothelial and subepithelial basement membrane was conserved in fibrotic mouse lungs and, additionally, we observed localization to the interstitial ECM as demonstrated by colocalization with extracellular collagen I (Fig. 2B, right-hand panel, higher magnification insert). Localization of Gpx3 to the interstitial ECM was also evident in more distal areas of fibrotic mouse lung, even if less prominent (Supplemental Figure S1).

### High GPX3 levels are found in BALF of some HP and sarcoidosis, but not of IPF and SRIF patients

**Figure 3:**
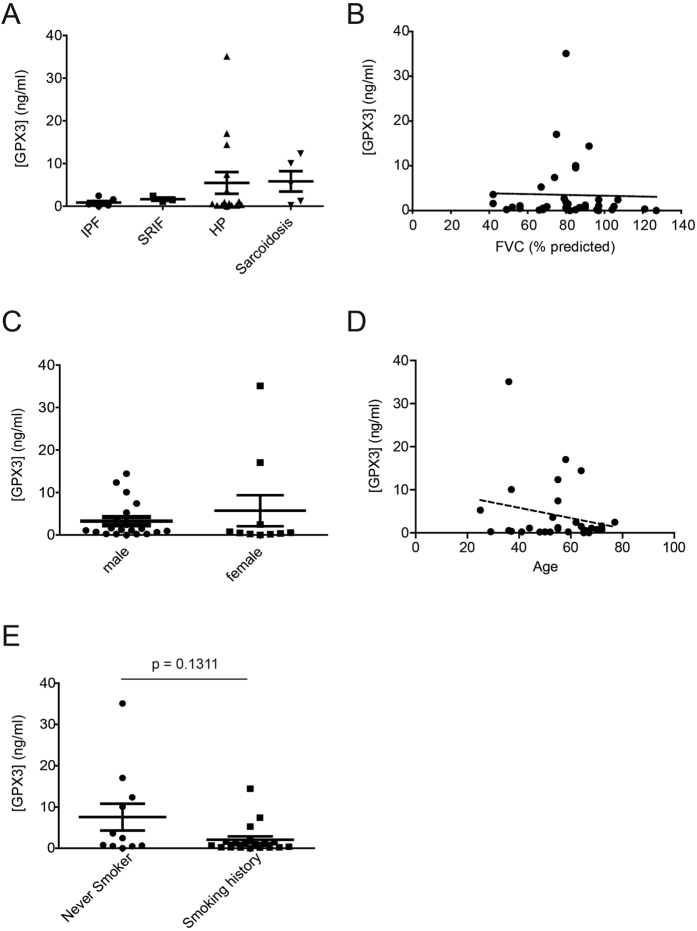
Increased GPX3 levels are detected in BALF from some patients with HP and sarcoidosis, but do not significantly correlate with lung function, gender, age, or smoking history. (**A**) GPX3 protein levels in BALF from patients with various ILD (n = 30) including IPF (n = 7), SRIF (n = 3), HP (n = 15), and sarcoidosis (n = 5) as measured by ELISA. GPX3 levels did not correlate with FVC (**B**), nor gender (**C**), nor age (**D**), nor the patients’ smoking history (**E**). For the latter, a non-significant trend for higher levels in never smokers is observed.

ELISA-based analysis of GPX3 levels in BALF from ILD patients showed consistently low GPX3 levels in patients with IPF and SRIF, but high levels for some HP and sarcoidosis patients (Fig. 3A). In contrast to the animal lung function data (*cf*. Fig. 1D), there was no correlation of BALF GPX3 levels with forced vital capacity (FVC) (Fig. 3B). As gender-specific differences in serum GPX3 and selenoprotein status have been observed^40^,^41^, we examined the relationship between gender and GPX3 levels in all BALF samples, but found no significant influence of gender on GPX3 levels in this cohort (Fig. 3C). Given that GPX3 is a selenoprotein and selenium deficiency has been associated with aging and increased mortality among the elderly^42^,^43^,^44^, we also examined a possible correlation between age and GPX3 levels and found that there was no significant correlation (Fig. 3D). Finally, smoking status also did not have a significant effect, although, interestingly, GPX3 tended to be decreased in BALF of patients with smoking history (Fig. 3E).

### GPX3 is upregulated in total tissue lysates from IPF patients

**Figure 4:**
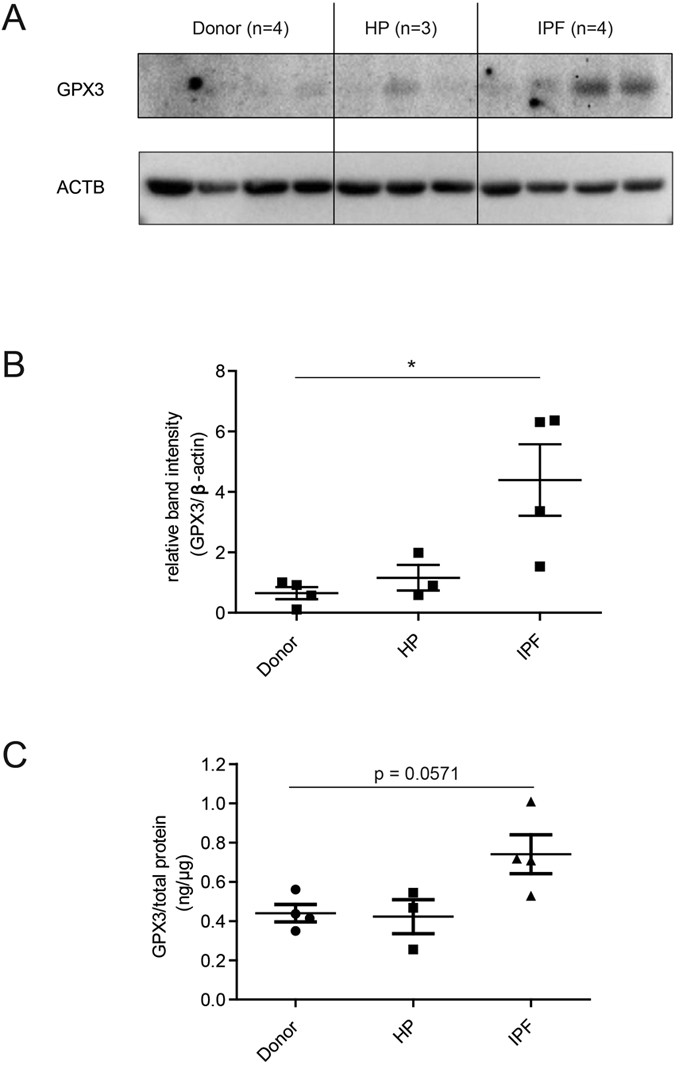
GPX3 protein is upregulated in total tissue homogenate from IPF lung. (**A**) Western Blot analysis of total lung tissue homogenate showed upregulation of GPX3 in patients with IPF (n = 4), but not HP (n = 3), relative to donor samples (n = 4). (**B**) Densitometric analysis of the Western Blot showed that GPX3 upregulation in IPF is significant. (**C**) ELISA using an independent antibody confirms upregulation of GPX3 in the same samples. Data shown are mean ± SEM and two-tailed Mann-Whitney test was used for statistical analysis (*p < 0.05). ACTB, β-actin as loading control.

Western Blot analysis and ELISA, using independently derived antibodies, showed upregulation of GPX3 protein levels in total lung lysates from IPF patients, but not from end-stage HP patients, relative to healthy donor control (Fig. 4, Supplementary Figure S2).

### In lung tissue sections of HP and IPF patients, GPX3 localizes to ECM structures

**Figure 5:**
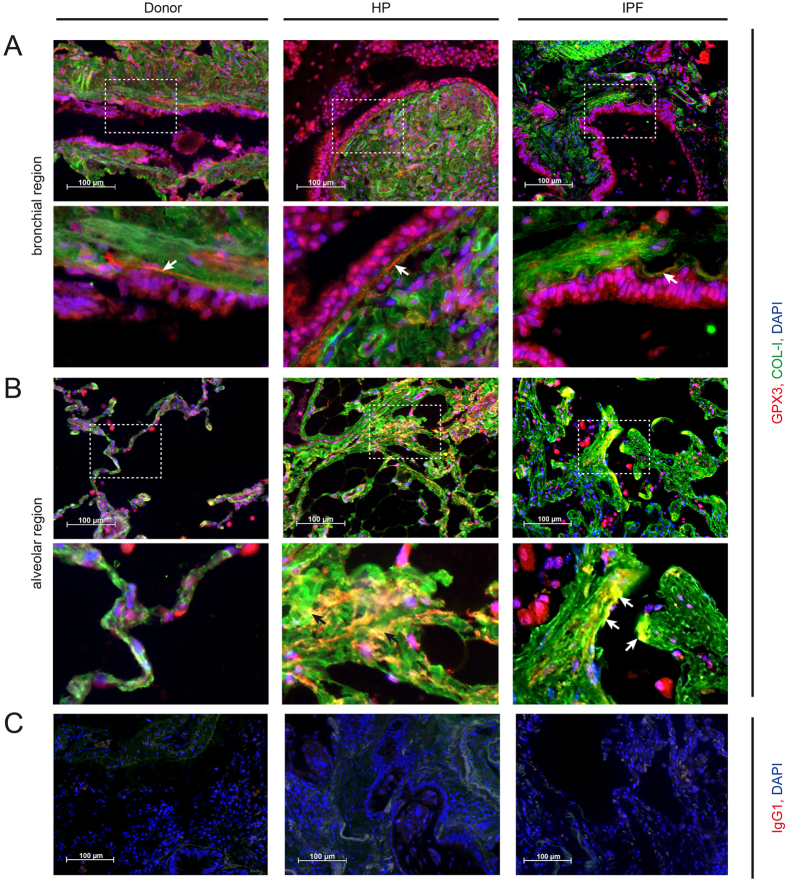
GPX3 is secreted by bronchial epithelial cells and localizes to ECM structures in donor, HP and IPF patients. Representative immunofluorescence analysis of donor, HP and IPF patients tissue with GPX3 (red), COL-I (green), and DAPI (blue) are depicted from bronchial (**A**) and alveolar regions (**B**) together with an isotype control for GPX3 staining (**C**). Scale bar: 100 μm. Arrows indicate co-localization of GPX3 with ECM structures: Subepithelial basement membrane (**A**, higher magnification inserts) and fibrotic interstitial ECM (**B**, higher magnification inserts).

Immunofluorescence analysis of human tissue sections supported the colocalization of GPX3 with the bronchial basement membrane in donor, HP and IPF patients, as demonstrated by costaining with extracellular collagen type I (Fig. 5A, indicated with arrows in the higher magnification inserts). Bronchial epithelial cells and interstitial cells, next to not further characterized immune cells, seem to be major producers of GPX3 in the human lung. Additionally, in contrast to donor tissue, GPX3 clearly also localized to the interstitial matrix in HP and IPF patients (Fig. 5B, indicated with arrows in the higher magnification inserts).

### GPX3 is expressed by primary human bronchial epithelial cells and lung fibroblasts and is variably regulated by TGF-β1, TNF-α, and the oxidative stress inducer menadione

**Figure 6:**
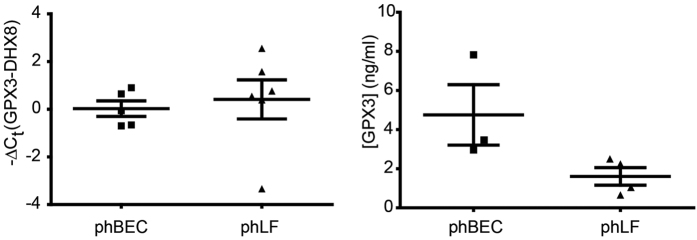
GPX3 is expressed by differentiated primary human bronchial epithelial cells (phBEC) and primary human lung fibroblasts (phLF) in culture. Left panel: Baseline GPX3 transcript levels in phBEC and phLF normalized to DHX8 transcript levels. Right panel: GPX3 protein levels in cell culture supernatant as measured by ELISA.

Next, we assessed whether GPX3 was expressed in primary human bronchial epithelial cells (phBECs) and primary human lung fibroblasts (phLFs) *in vitro*. For both cell types we observed similar expression of GPX3 on transcript level, comparable to expression of ATP-dependent RNA helicase DHX8 (DHX8) used as endogenous control^45^ (Fig. 6, left-hand panel). GPX3 was also detected in the cell culture supernatant of both cell types (Fig. 6, right-hand panel). Here, GPX3 levels were higher in the basolateral cell culture supernatant of phBECs than in cell culture supernatant of phLF.

**Figure 7:**
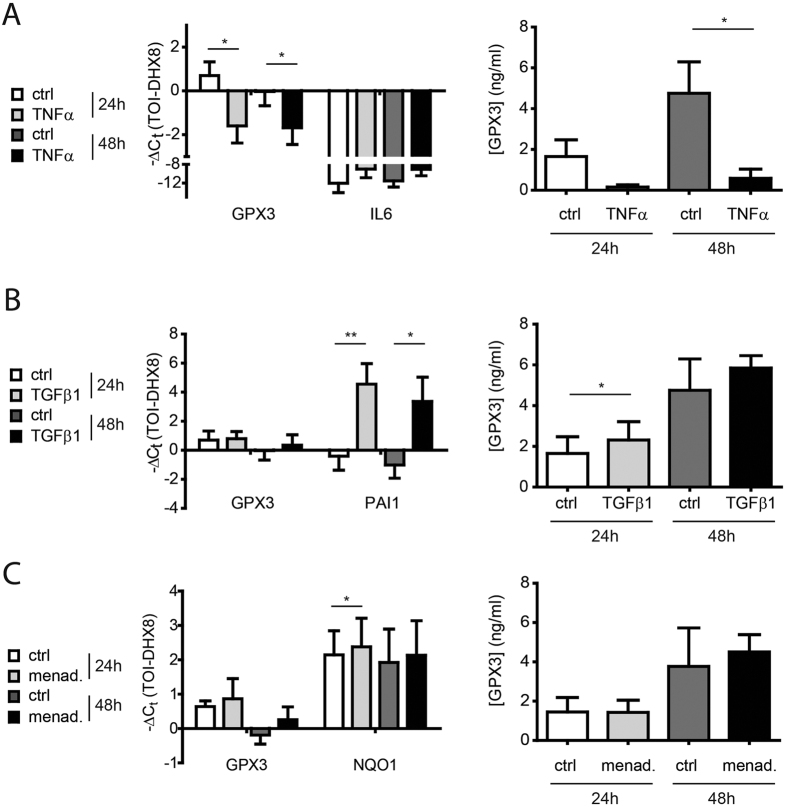
Expression of glutathione peroxidase 3 (GPX3) is downregulated by TNF-α, moderately upregulated by TGF-β1, but not consistently regulated by the oxidative stress inducer menadione in differentiated primary human bronchial epithelial cells (phBEC). (**A**) Left panel: GPX3 transcript levels in differentiated phBEC after treatment with 10 ng/ml TNF-α for 24 and 48 hours. Interleukin-6 (IL6) transcript is used as a positive control for TNF-α activity. Right panel: GPX3 protein levels in cell culture supernatant in response to TNF-α, as measured by ELISA. (**B**) Left panel: GPX3 transcript levels in differentiated phBEC after treatment with 2 ng/ml TGF-β1 for 24 and 48 hours. Plasminogen activator inhibitor 1 (PAI1) transcript is used as a positive control for TGF-β1 activity. Right panel: GPX3 protein levels in cell culture supernatant in response to TGF-β1, as measured by ELISA. (**C**) Left panel: GPX3 and NQO1 transcript levels in differentiated phBEC after treatment with 10 μM menadione for 24 and 48 hours. Right panel: GPX3 protein levels in cell culture supernatant in response to menadione, as measured by ELISA. The presented data are based on three independent experiments (phBEC isolated from three different donors) and given as mean ± SD. Statistical analysis was performed using paired t-test. *p < 0.1; **p < 0.01; ***p < 0.001.

**Figure 8:**
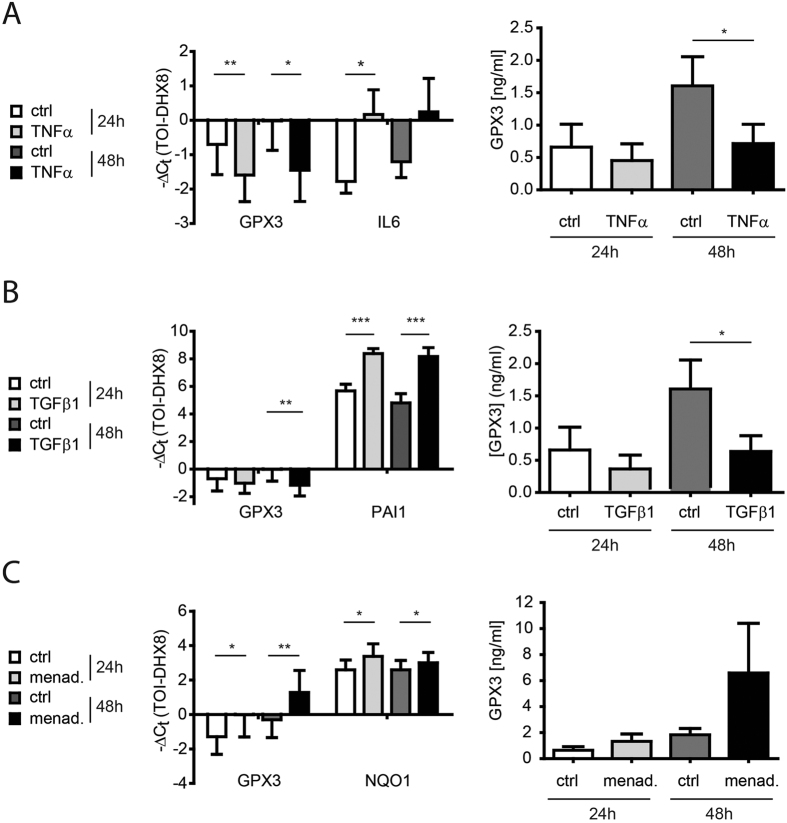
Expression of glutathione peroxidase 3 (GPX3), is downregulated by TGF-β1 and TNF-α, but upregulated by the oxidative stress inducer menadione in primary human lung fibroblasts (phLF). (**A**) Left panel: GPX3 transcript levels in phLF after treatment with 10 ng/ml TNF-α for 24 and 48 hours. Interleukin-6 (IL6) transcript is used as a positive control for TNF-α activity. Right panel: GPX3 protein levels in cell culture supernatant in response to TNF-α, as measured by ELISA. (**B**) Left panel: GPX3 transcript levels in phLF after treatment with 2 ng/ml TGF-β1 for 24 and 48 hours. Plasminogen activator inhibitor 1 (PAI1) transcript is used as a positive control for TGF-β1 activity. Right panel: GPX3 protein levels in cell culture supernatant in response to TGF-β1, as measured by ELISA. (**C**) Left panel: GPX3 and NQO1 transcript levels in differentiated phBEC after treatment with 10 μM menadione for 24 and 48 hours. Right panel: GPX3 protein levels in cell culture supernatant in response to menadione, as measured by ELISA. The presented data are based on four independent experiments (phLF isolated from four different donors) and given as mean ± SD. Statistical analysis was performed using paired t-test. *p < 0.1; **p < 0.01; ***p < 0.001.

As we had observed upregulation of GPX3 both during the inflammatory (day 7) and fibrotic (day 14) phase of bleomycin-induced lung fibrosis^35^,^46^ (Fig. 1B,C,E), upregulation in IPF (Fig. 4), as well as high levels in BALF from ILDs with a strong inflammatory component such as HP and sarcoidosis (*cf*. Fig. 3A), we investigated whether GPX3 is upregulated by the proinflammatory cytokine TNF-α or the profibrotic cytokine TGF-β1 in phBEC and phLF. We found consistent downregulation of GPX3 expression by TNF-α in both cell types, both on transcript and on secreted protein level (Figs 7A and 8A). For TGF-β1, we found a weak upregulation in phBEC (Fig. 7B), but significant downregulation in phLF (Fig. 8B). In contrast, menadione, an inducer of oxidative stress, increased GPX3 expression in phLF, but not in phBEC (Figs 7C and 8C, respectively). Efficacy of TNF-α, TGF-β1, and menadione treatment was verified using well-established target genes, *i.e.* interleukin-6 (IL6), plasminogen activator inhibitor 1 (PAI1), and NAD(P)H dehydrogenase [quinone] 1 (NQO1), respectively.

## Discussion

Many ILDs are triggered by oxidative insults to the airways, but regulation of extracellular antioxidant proteins has gained little attention in this context. In the present study, we show that levels of the antioxidant protein GPX3 are increased in BALF and tissue during bleomycin-induced lung fibrosis, as well as in BALF of some HP and sarcoidosis patients and in total lung homogenates of IPF patients. GPX3 was expressed by bronchial epithelial cells and interstitial fibroblasts. While GPX3 localized mainly to the basement membrane of the bronchial epithelium and endothelium in healthy mouse and human tissue, we found localization also to the interstitial ECM in fibrotic tissue compartments. Finally, GPX3 expression in bronchial epithelial cells and fibroblasts was downregulated by TNF-α, but upregulated in a cell-type-specific manner by TGF-β and menadione, respectively.

As one of the lung-abundant extracellular antioxidant proteins^33^, GPX3 has the potential to fulfil an important protective function in the lung, but regulation and localization of GPX3 in ILD has received little attention. The mouse model of bleomycin-induced lung fibrosis is typically used as a model for ILD. Using this model we found that Gpx3 was expressed by bronchial epithelial cells, secreted in active form into the ELF, and upregulated in lung fibrosis both in BALF and in lung tissue. In samples from IPF patients, we found significant upregulation of GPX3 in total tissue homogenates. Furthermore, we could show that primary human bronchial epithelial cells und fibroblasts express GPX3 in culture. This is in agreement with a number of previous studies that showed expression of GPX3 by bronchial epithelial cells and presence in the ELF^36^,^47^. More recent studies employing immunofluorescent stainings, however, failed to show expression in bronchial epithelial cells and fibroblasts, and presence of Gpx3 in the lung was mostly attributed to serum Gpx3 supplied primarily by the kidneys^32^,^33^,^48^. Our data highlight that GPX3 is expressed and secreted by mouse and human bronchial epithelial cells already under physiological conditions and increased in mouse and human lung fibrosis.

In mouse and human lung tissue sections, our results demonstrate localization of GPX3 to the subepithelial and subendothelial basement membrane, in agreement with previous studies showing association of GPX3 with the basement membrane^32^,^33^,^48^. It has been proposed that also this basement membrane-associated GPX3 is mostly kidney-derived^32^, whereas our results suggest that bronchial epithelial cells secrete GPX3 and thus contribute to the subepithelial basement membrane-associated population. This is further underlined by the detection of GPX3 in ELF following lung injury, both in the mouse model of bleomycin-induced lung fibrosis and in ILD, in particular in HP and sarcoidosis. However in our small cohort, GPX3 levels were neither significantly associated with a particular ILD nor correlated with lung function, as proposed by the animal study. However, clearly a larger patient cohort would be necessary to ultimately rule out possible correlations.

Importantly, we show, to our knowledge for the first time, that in bleomycin-induced lung fibrosis, HP, and IPF, GPX3 additionally localizes to the interstitial matrix in lung fibrosis. This raises the possibility that GPX3 might protect interstitial fibrotic ECM from oxidative damage with a potential impact on the resolvability of fibrotic matrix. Hence, the function of ECM-bound GPX3 clearly warrants further investigation.

In IPF patients, interestingly, GPX3 levels were significantly increased in total lung homogenate, but consistently low in BALF. This might argue for an altered compartmentalization of GPX3 in IPF, *e.g.* a specific increase of fibroblast-specific *GPX3* expression or a spatial shift from apically secreted soluble GPX3 to interstitial matrix-associated GPX3, reminiscent of what has been observed in a mouse model of influenza pneumonia^33^.

In the mouse model of bleomycin-induced lung fibrosis, Gpx3 levels in tissue and BALF were increased in the inflammatory (day 3–7) and fibrotic (day 14–28) phase^35^,^46^, but had returned to baseline after resolution of fibrosis at day 56. Levels of Gpx3 as well as Gpx activity in BALF were highest in the inflammatory phase (day 7) of bleomycin-induced lung fibrosis, suggesting upregulation by inflammatory rather than profibrotic mediators (*cf*. Fig. 1B,C,E). Also, GPX3 has been found upregulated in the ELF of individuals with asthma and chronic beryllium disease, the latter an ILD characterized by granulomatous lung inflammation, similar to sarcoidosis^47^,^49^. Equally, our own data show higher levels of GPX3 in ILD typically associated with an inflammatory component (HP, sarcoidosis) than in ILD which are considered less associated with inflammation as IPF and SRIF (*cf*. Fig. 3). This lead us to investigate whether TNF-α as a classical proinflammatory cytokine and TGF-β as central profibrotic stimulus regulated GPX3 expression in primary human bronchial epithelial cells and fibroblasts.

In both lung cell types TNF-α strongly suppressed GPX3 expression. This was an unexpected result insofar that GPX3 is considered a classical Nrf2-induced gene and TNF-α has repeatedly been reported to upregulate other Nrf2 target genes in various cell types including leukocytes of the myeloid lineage, vascular endothelial cells, and osteoblasts^50^,^51^,^52^,^53^,^54^. However, to our knowledge, only one study has assessed the effect of TNF-α on GPX3 expression and shown that, in adipose tissue, GPX3 expression is markedly decreased by TNF-α, in agreement with our observation^55^.

This is the first study to show that GPX3 is regulated by TGF-β in a cell-type-specific manner. TGF-β treatment led to a moderate upregulation of GPX3 secretion in phBEC, but to downregulation of GPX3 on both transcript and protein level in phLF. TGF-β has been shown to inactivate Nrf2 and repress expression of various Nrf2 target genes in both mesenchymal cells and epithelial cells^56^,^57^, but also upregulation of antioxidant genes by TGF-β has been reported^58^,^59^. Our results emphasize the importance of the cellular context for modulation of the oxidative stress response by TGF-β.

Interestingly, menadione appeared to be more effective as inducer of the oxidative stress response in phLF than in phBEC. 10 μM menadione, a concentration which has been shown to upregulate GPX3 transcription in an immortalized bronchial epithelial cell line^60^, consistently induced expression of both GPX3 and NQO1 in phLF while the effects in phBEC were more variable.

In summary, our *in vitro* results suggest, that TGF-β and oxidative stress contribute to increased GPX3 expression in lung fibrosis in a cell-type-specific manner, upregulating GPX3 in bronchial epithelial cells and interstitial fibroblasts, respectively. In contrast, our findings uniformly rule out TNF-α as positive regulator of GPX3 in ILD. Clearly, other factors important for ILD pathogenesis, not assessed in the present study, might play a role in regulation of GPX3 expression, as *e.g.* hypoxia, other proinflammatory mediators, or cigarette smoke^49^,^61^. As to the latter, it is worth mentioning that in all ILD patients assessed in this study, there was a non-significant trend (*cf*. Fig. 3E) for never-smokers to exhibit higher GPX3 levels in BALF than patients with a smoking history. Similarly, albeit also in a small cohort, Comhair *et al*. reported that never-smoking patients with chronic beryllium disease (CBD) displayed higher extracellular GPX activity in BALF than ex-smoking CBD patients^49^.

In conclusion, we demonstrate that, under normal conditions, GPX3 is expressed and secreted in active form into the ELF by bronchial epithelial cells. In addition to localization to basement membranes reported previously, GPX3 can be expressed by lung fibroblasts and associate with interstitial fibrotic ECM, suggesting a protective anti-oxidant function in this compartment. Our findings further indicate that TGF-β and oxidative stress, but not TNF-α, contribute to upregulation of GPX3 in lung fibrosis in a cell-type-specific manner.

## Materials and Methods

### Animals

Pathogen-free female C57BL/6 mice (10–12 weeks old) were obtained from Charles River and housed in rooms maintained at constant temperature and humidity with a 12 hours light cycle. Animals were allowed food and water *ad libitum*. All animal experiments were conducted under strict governmental and international guidelines and were approved by the local government for the administrative region of Upper Bavaria, Germany.

### Induction and measurement of murine pulmonary fibrosis

Pulmonary fibrosis was induced in female C57BL/6 mice (10–12 weeks old) by a single intratracheal instillation of 50 μl of bleomycin (3 U/kg, Sigma Aldrich, Taufkirchen, Germany) dissolved in sterile saline, and applied using the MicroSprayer Aerosolizer, Model IA-1 C (Penn-Century, Wyndmoor, PA). Control mice were instilled with 50 μl of saline. After instillation, mice were kept for 3, 7, 14, 28, and 56 days. Before sacrifice, mice were anesthetized with ketamine/xylazine followed by lung function measurement and tissue harvesting as previously described^35^,^62^. Bronchoalveolar lavage fluid (BALF) was obtained as previously described^35^. Fibrosis was further assessed by bronchoalveolar lavage cell counts and histology evaluation.

### Proteomic data

Time- and compartment-resolved protein profiles from BALF and lung tissue during bleomycin-induced lung fibrosis were extracted from proteomics data generated previously by us^35^. These data are publicly available in the supplementary material of the corresponding open access article^35^ or via the MaxQB database (http://maxqb.biochem.mpg.de/mxdb/ project/show/P013)^63^. Data are always reported as log2 fold changes relative to the median of all control mice from the study (n = 28), if not mentioned otherwise.

### Patients

Patients diagnosed with IPF (n = 7), SRIF (n = 3), HP (n = 15), and sarcoidosis (n = 5) were included in the study after signing an informed consent agreement. Patient demographics und lung function data are provided in Table 1. The study was approved by the Ethics Committee of Thomayer Hospital and the Institute of Clinical and Experimental Medicine, Prague, Czech Republic and carried out in accordance with the approved guidelines. All enrolled patients provided a medical history that included information on smoking and potential exposure to inhaled antigens and underwent a medical examination that involved: a chest X-ray, HRCT of the chest, pulmonary function tests, and bronchoscopy with bronchoalveolar lavage (BAL). IPF was diagnosed according to the ERS/ATS guidelines^64^. SRIF was diagnosed according to typical radiologic and histopathological findings in combination with a history of smoking, as published by Katzenstein *et al*.^3^. Diagnosis of HP was performed as reported previously^65^. Finally, sarcoidosis was diagnosed using the ATS/ERS/WASOG criteria^66^. Notably, this patient cohort included HP and sarcoidosis patients at different disease stages including such with resolving disease.

For immunofluorescence stainings and Western Blot analysis, resected human lung tissue and lung explant material was obtained from the BioArchive CPC-M for lung diseases at the Comprehensive Pneumology Center (CPC). Here, all HP and IPF samples were lung explant material from transplantations, reflecting non-resolving end-stage disease. All participants gave written informed consent and this part of the study was approved by the local ethics committee of Ludwig-Maximilians University of Munich, Germany (333-10) and carried out in accordance with the approved guidelines.

### Pulmonary function tests

Forced vital capacity (FVC) was obtained using a ZAN 100 Flowhandy II (Inspire, Oberthulba, Germany). Values are shown as percentage of the predicted value.

### Bronchoalveolar lavage (BAL)

BAL was performed during fiberoptic bronchoscopy under local anaesthesia. The bronchoscope was wedged into a segmental bronchus of the middle lobe. Five fractions of 50 ml of lukewarm saline were instilled and, after each instillation, gently aspirated. Only samples with a recovery > 50% were used (mean recovery per one fraction was 57 ± 20%). The retrieved fluid was placed in a sterile container before division for further investigation. Five millilitres of BALF was aliquoted and stored at −80 °C.

### Human lung material and isolation and culture of primary human lung fibroblasts

For isolation of primary human lung fibroblasts (phLF), for Western Blot, and for immunofluorescent stainings, resected human lung tissue and lung explant material was obtained from the BioArchive CPC-M for lung diseases at the Comprehensive Pneumology Center (CPC). HP and IPF patient material was exclusively derived from lung explant material, representing end-stage disease. Primary human lung fibroblasts for *in vitro* experiments were isolated from histologically normal regions adjacent to resected lung tumours. All participants gave written informed consent and the study was approved by the local ethics committee of Ludwig-Maximilians University of Munich, Germany (333-10).

Isolation of phLF from lung tissue was performed as described in detail elsewhere^46^: Briefly, human lung specimens were dissected and digested using 1 mg/ml Collagenase I (Biochrom, Cambridge, UK) at 37 °C for 1 hour. Samples were then passed through nylon filters with a pore size of 70 μm (BD Falcon, Franklin Lakes, NJ, USA), followed by centrifugation at 400 × *g* and 4 °C for 5 minutes. Pellets were resuspended in DMEM/F-12 medium (Life Technologies, Carlsbad, CA, USA) supplemented with 20% foetal bovine serum (FBS, Pan Biotech, Aidenbach, Germany) and penicillin/streptomycin (Life Technologies) and plated on 10 cm cell-culture dishes. Medium was changed after 2 days and cells were split after reaching a confluence of 80–90%. For the present study, phLF from four different donors were used in passages 6–8. Fibroblasts were cultured in DMEM/F12 (Life Technologies) supplemented with 20% FBS (Pan Biotech) and penicillin/streptomycin.

### Culture and differentiation of primary human bronchial epithelial cells (phBEC)

Normal primary human bronchial epithelial cells (phBEC) from three healthy donors were obtained from Lonza (Wokingham, UK), cultured in BEGM medium (Lonza) and differentiated at the air-liquid interface (ALI) in PneumaCult™-ALI medium (Stemcell Technologies, Cologne, Germany) for up to 28–33 days as described previously^67^. Briefly, 80.000–90.000 cells (passage 2) were seeded on human placental collagen type IV-coated (Sigma-Aldrich; St. Louis, MO) 12-well transwell inserts (clear, 0.4 μm; Corning; New York, NY, USA) in BEGM medium. Cells were air-lifted upon confluency (=day 0 of ALI culture) by removing the apical medium and substituting the basolateral medium with PneumaCult™-ALI medium containing 1% penicillin/streptomycin. Medium was changed every other day and cells were washed apically with HBSS (Life Technologies; Carlsbad, CA, USA) once a week to remove mucus.

### Treatment of phLF and phBEC with TGF-β1, TNF-α, and menadione

For treatment of phLF, cells were seeded at a density of approximately 20,000 cells/cm^2^, starved for 24 hours in DMEM/F12 with 0.5% FBS and 100 nM sodium selenite, followed by treatment with 2 ng/ml TGF-β1 (R&D Systems, Minneapolis, MM, USA), 10 ng/ml TNF-α (PeproTech, Rocky Hill, NJ, USA), and 10 μM menadione (Sigma-Aldrich) in starvation medium for the indicated time points.

For treatment of phBEC, fully differentiated cells were stimulated for 24 or 48 hours with 2 ng/ml TGF-β1, 10 ng/ml TNF-α, 10 μM menadione from the apical (in 100 μl HBSS) and basolateral side (in 1 ml ALI medium) simultaneously. Menadione was dissolved in dimethylsulfoxide (DMSO); here, control cells were treated using the same volume of DMSO.

### RNA isolation and real-time quantitative reverse-transcriptase PCR (qRT-PCR) analysis

For RNA extraction, the peqGold RNA isolation kit (Peqlab, Erlangen, Germany) was used for phLF and the RNeasy Mini Plus Kit (Quiagen; Venlo, Holland) was used for phBEC according to the manufacturer’s instructions. RNA was reverse-transcribed in a 40 μl reaction using M-MLV reverse transcriptase and random hexamers, according to the manufacturer’s protocol (Life Technologies). Quantitative real-time PCR (qRT-PCR) was performed using SYBR Green PCR master mix (Roche Applied Science, Mannheim, Germany).

Relative transcript abundance of a gene is expressed as −ΔC_t_ values (−ΔC_t_ = C_t_^reference^ − C_t_^target^) or as fold change derived from the relevant ΔΔC_t_ values, using 2^−(ΔΔCt)^. For specific gene amplification, primers listed in Table 2 were used. ATP-dependent RNA helicase DHX8 (DHX8) and hypoxanthine-guanine phosphoribosyltransferase (HPRT) were used as endogenous controls for standardization of relative mRNA expression in phLF and phBEC, respectively. Normalization with both reference genes provided similar results and, for data presentation, analysis was performed using DHX8 as reference gene. In a previous study^45^ we have assessed the quality of a number of reference genes for lung transcriptomes derived from Genevestigator V3^68^ and found that DHX8 was a reliable reference gene in this context.

### Western Blot analysis for detection of GPX3 in whole lung lysates

Western Blot analysis was performed as described previously^46^. For detection of human GPX3, mouse monoclonal anti-GPX3 antibody (abcam, Cambridge, UK; ab27325) and for detection of β-actin, an HRP-conjugated anti-β-actin antibody (Sigma Aldrich, Louis, MO, USA; A3854) was used.

### ELISA for detection of GPX3 in BALF, cell culture supernatant, and tissue homogenates

For detection of human GPX3 in human BALF, cell culture supernatants and tissue homogenates, the GPX3 human ELISA Kit (AdipoGen, San Diego, CA, USA) was used according to manufacturer’s instructions. For phBEC, only the basolateral supernatant was analysed, as no GPX3 was detected in the apical supernatant. For BALF and cell culture supernatants, 100 μl sample were used and the results expressed as a concentration in ng/ml. For tissue homogenates, 5 μg total protein were used and the results normalized to amount of total input protein and expressed as ng GPX3/μg protein.

### Measurement of glutathione peroxidase activity in mouse BALF

Selenium-dependent glutathione peroxidase activity in mouse BALF was measured by monitoring fluorescence at 460 nm with an excitation wavelength of 355 nm in a Berthold plate reader (Tristar LB941, Bad Wildbad, Germany)^69^. Reactions were performed in 50 mM sodium phosphate buffer pH 7.0 containing 0.1% Triton-X-100, 0.40 mM EDTA, 0.20 mM NADPH, 0.25 U glutathione reductase from Baker’s yeast, 1 mM L-glutathione, and 100 μM *t*-butyl hydroperoxide (all Sigma-Aldrich) in a total volume of 200 μl. Reactions were started by adding 50 μl mouse BALF. 50 μl phosphate-buffered saline was used as negative and 0.01 U bovine GPX (Sigma Aldrich) as positive control, respectively. Data are reported as activity fold changes relative to the median of control activity for each time point (n = 29).

### Immunofluorescent stainings

For staining of tissue sections, human and murine lung tissue was fixed in 4% formalin prior to paraffin embedding. Three-micron sections were prepared and mounted on slides, followed by deparaffinization and immunofluorescent staining according to a standard protocol as follows: For deparaffinization, the paraffin-embedded sections were placed at 60 °C for at least 30 min, incubated twice in xylene (5 minutes each), and then transferred into 100% ethanol (2 minutes), 100% ethanol (2 minutes), 90% ethanol (1 minute), 80% ethanol (1 minute), and 70% ethanol (1 minute) at room temperature. Sections were rinsed in deionized water and stored in 0.5 M Tris, 1.5 M NaCl, pH 6.8 (termed Tris buffer hereafter). For antigen retrieval, slides were immersed in citrate buffer pH 6.0 and heated in a Decloaking Chamber 30 seconds at 125 °C, followed by 10 seconds at 90 °C. Then, the slides were allowed to slowly cool down to room temperature. Slides were washed three times in Tris buffer and primary antibody dilutions were prepared in antibody diluent (Zytomed Systems, Berlin, Germany), added to each tissue section in a wet chamber and incubated overnight. Primary antibodies used were mouse monoclonal anti-Gpx3 (Abcam, Cambridge, UK, 1:50 or 1:100) and rabbit polyclonal anti-Collagen I antibody (Rockland, Gilbertsville, PA, USA, 1:200). Secondary antibodies were Alexa Fluor 488 goat anti-mouse IgG or Alexa Fluor 568 goat anti-rabbit IgG from Life Technologies (both 1:400). Slides were rinsed three times with Tris buffer and counterstained with DAPI (Sigma-Aldrich, 1:2500) for 1 minute in darkness. Then slides were rinsed three times with Tris buffer, covered with Fluorescence Mounting Medium (Dako, Hamburg, Germany) and examined under an Axio Imager Microscope (Carl Zeiss, Jena, Germany).

## Additional Information

**How to cite this article**: Schamberger, A. C. *et al*. Glutathione peroxidase 3 localizes to the epithelial lining fluid and the extracellular matrix in interstitial lung disease. *Sci. Rep.* **6**, 29952; doi: 10.1038/srep29952 (2016).

## Supplementary Material

Supplementary Information

## Figures and Tables

**Table 1:** Patient demographics and pulmonary function test results.

	**IPF (n = 7)**	**SRIF (n = 3)**	**HP (n = 15)**	**Sarcoidosis (n = 5)**
Age (mean ± SD)	69 ± 6	57 ± 11	52 ± 11	40 ± 14
Female sex	5	0	5	—
No smoking history	4	0	5	2
DLCO (mean ± SD, % predicted)	48 ± 13	58 ± 19	46 ± 27	74 ± 17
FEV1 (mean ± SD, % predicted)	82 ± 21	90 ± 9	72 ± 21^1^	85 ± 8
FVC (mean ± SD, % predicted)	78 ± 15	95 ± 13	78 ± 24^1^	89 ± 15

^1^n = 13, values missing for two patients.

**Table 2:** Primers used for qRT-PCR.

**Target**	**Forward primer (5′-3′)**	**Reverse primer (5′-3′)**
*DHX8*	TGACCCAGAGAAGTGGGAGA	ATCTCAAGGTCCTCATCTTCTTCA
*GPX3*	TTGATGGGGAGGAGTACATCC	AGACCGAATGGTGCAAGCTC
*HPRT*	AAGGACCCCACGAAGTGTTG	GGCTTTGTATTTTGCTTTTCCA
*IL6*	TCTTTTGGAGTTTGAGGTAT	CATCTAGATTCTTTGCCTTT
*NQO1*	AAGGACATCACAGGTAAACT	GAACTGGAATATCACAAGGT
*PAI1*	GACATCCTGGAACTGCCCTA	GGTCATGTTGCCTTTCCAGT

Primers were synthesized by MWG Eurofins (Ebersberg, Germany).
