# Supplementary material for: Glutathione peroxidase 3 localizes to the epithelial lining fluid and the extracellular matrix in interstitial lung disease
Source: Sci Rep. 2016 Jul 20;6:29952. doi: 10.1038/srep29952 (PMC4951690; doi:10.1038/srep29952)
Supplement: Supplementary Information [file srep29952-s1.pdf]

ONLINE DATA SUPPLEMENT

GLUTATHIONE PEROXIDASE 3 LOCALIZES TO THE EPITHELIAL LINING FLUID AND THE EXTRACELLULAR MATRIX IN INTERSTITIAL LUNG DISEASE

Andrea C. Schamberger[1], Herbert B. Schiller[1,2], Isis E. Fernandez[1], Martina Sterclova[3], Katharina Heinzelmann[1], Elisabeth Hennen[1], Rudolf Hatz[4,5], Jürgen Behr[5,6], Martina Vašáková[3], Matthias Mann[2], Oliver Eickelberg[1], and Claudia A. Staab-Weijnitz[1*]

[1]Comprehensive Pneumology Center, Helmholtz Zentrum München, Munich, Germany; Member of the German Center of Lung Research (DZL)

[2] Department of Proteomics and Signal Transduction, Max-Planck Institute of Biochemistry, Martinsried, Germany

[3]Department of Pneumology, Thomayer Hospital, Prague 4-Krč, 140 59, Czech Republic

[4]Thoraxchirurgisches Zentrum, Klinik für Allgemeine-, Viszeral-, Transplantations-, Gefäß- und Thoraxchirurgie, Klinikum Großhadern, Ludwig-Maximilians-Universität, Munich, Germany;

[5]Asklepios Fachkliniken München-Gauting, Munich, Germany;

[6]Medizinische Klinik und Poliklinik V, Klinikum der Ludwig-Maximilians-Universität, Munich, Germany

[*]To whom correspondence should be addressed: Claudia Staab-Weijnitz, Comprehensive Pneumology Center, Ludwig-Maximilians-Universität and Helmholtz Zentrum München, Max-Lebsche-Platz 31, 81377 München, Germany, Tel.: 0049(89)31874681; Fax: 0049(89)31874661; Email: staab-weijnitz@helmholtz-muenchen.de

SUPPLEMENTARY FIGURE LEGENDS

Supplementary figure S1: **Gpx3 also localizes to ECM structures in distal fibrotic areas of bleomycin (Bleo) instilled mouse lung.** Immunofluorescence analysis of Bleo mouse lungs at day 14 after instillation. Representative images with Gpx3 (red), Col-I (green), and DAPI (blue) are shown for distal alveolar regions. Scale bar: 100 µm. White arrows in the higher magnification inserts (lower panels) indicate colocalization of Gpx3 with interstitial ECM structures.

Supplementary figure S2: **GPX3 protein is increased in IPF total lung homogenates relative to donor control samples.** Western Blot analysis of GPX3 in donor, HP, and IPF lung tissue homogenate show upregulation of GPX3 (expected molecular weight after removel of signal peptide 23.8 kDa). Signals were documented with the ChemiDoc XRS+ Imaging System and merged with a photograph of the blot, so that the image here also shows the prestained molecular weight markers.

Figure S1

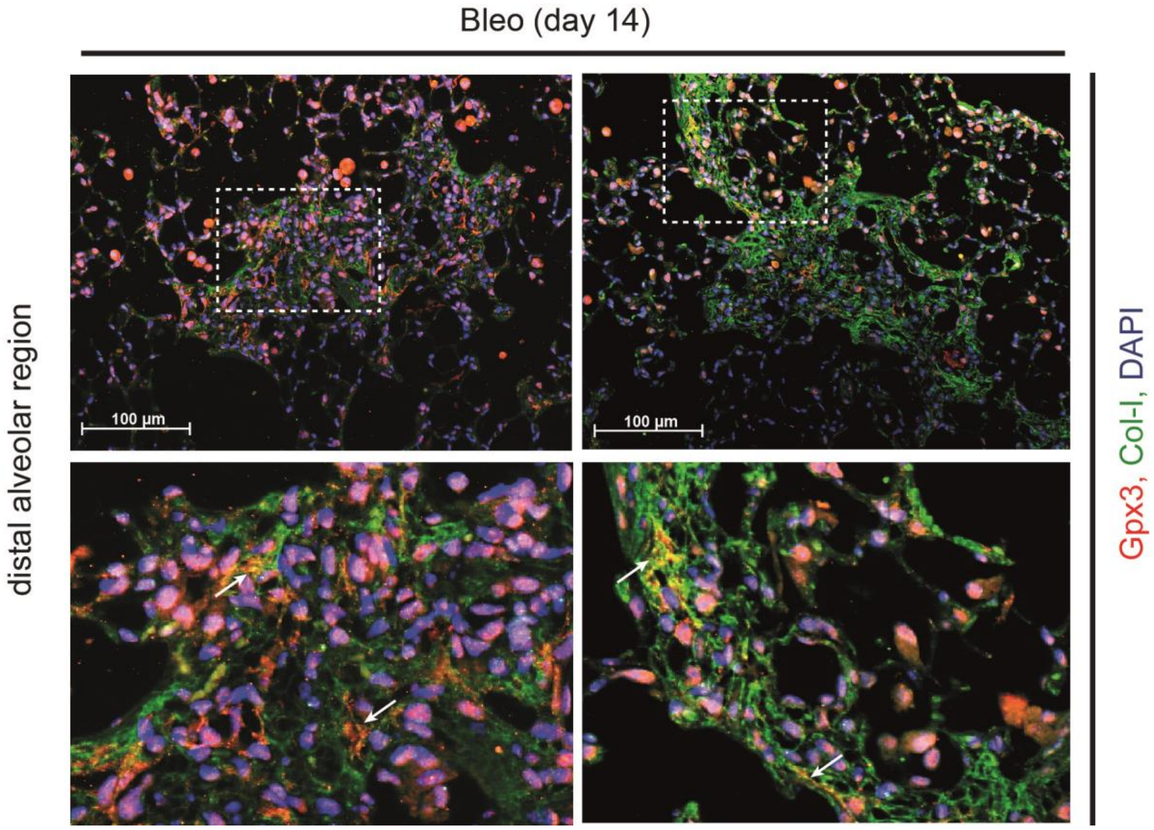

Bleo (day 14)
distal alveolar region
100 µm
100 µm
Gpx3, Col-I, DAPI

Figure S2

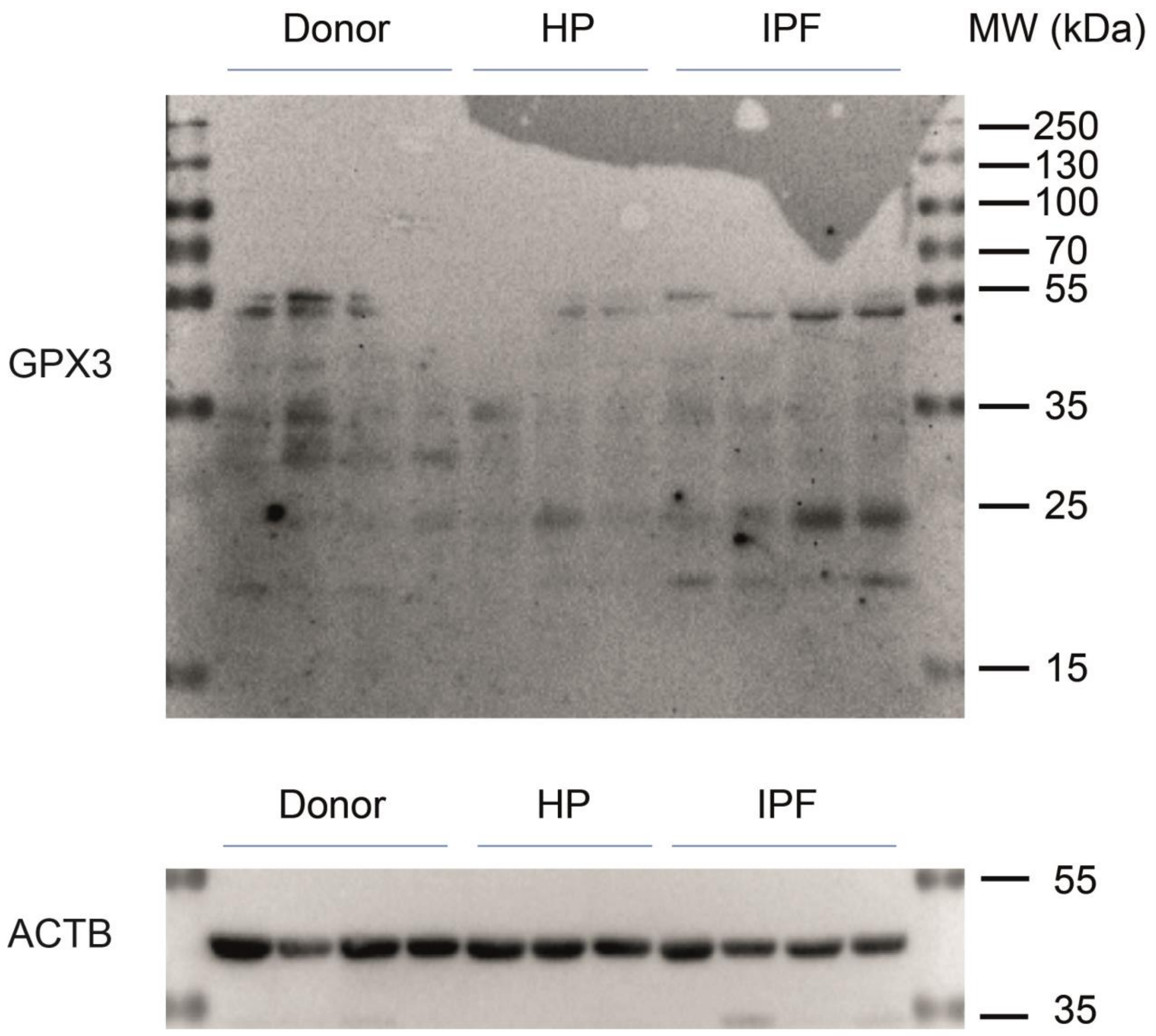
Donor
HP
IPF
MW (kDa)
GPX3
250
130
100
70
55
35
25
15
Donor
HP
IPF
ACTB
55
35
